# Restoring Fertility for Novel Interspecific Hybrids between *Kalanchoe garambiensis* and *K. nyikae* Using Colchicine Treatment

**DOI:** 10.3390/plants10020209

**Published:** 2021-01-22

**Authors:** Yi Kuang, Chi-Hsuan Lu, Fu-Chiun Hsu

**Affiliations:** Department of Horticulture and Landscape Architecture, National Taiwan University, Taipei 10617, Taiwan; r05628126@ntu.edu.tw (Y.K.); r08628105@ntu.edu.tw (C.-H.L.)

**Keywords:** *Kalanchoe*, interspecific hybridization, hybrid sterility, colchicine

## Abstract

Interspecific hybridization is an effective strategy in *Kalanchoe* breeding programs for the introduction of new traits. Wild species within the *Kalanchoe* genus are valuable genetic resources for providing new horticulture traits and to improve environmental adaptations. However, reproductive barriers associated with fertilization and hybrid sterility must be overcome to produce fertile hybrid progenies. To approach the breeding objectives for *Kalanchoe* cut flower cultivars with long stem traits and adaptation to tropical/subtropical regions, a tropical species endemic to Taiwan, *Kalanchoe garambiensis* Kudo, was used as a parent to cross with other long stem *Kalanchoe* species. Reciprocal crossing was effective in overcoming interspecific unilateral incompatibility in our crossed pairs. One superior hybrid, ‘103-1’, produced capsules without seeds by selfing and backcrossing with pollens from either parent. Other than the seedless trait, failure of pollen releasing from anther, pollen aggregation and no pollen germination in ‘103-1’ suggested its F_1_ sterility. Colchicine treatments on apical buds of ‘103-1’ successfully overcame potential meiotic abnormalities by doubling ploidy. For the first time, fertile interspecific hybrids of *K. garambiensis* and *K. nyikae* Engler were generated. The fertile hybrid has further produced progeny populations by crossing with *K. nyikae* or *K. blossfeldiana* von Poelln, ‘Ida’.

## 1. Introduction

*Kalanchoe* is the second most important house plant, with 94–100 million plants worth 67–69 million euros sold per year in international markets [1]. Broad *Kalanchoe* varieties and their long-lasting flowers provide customers with a wide spectrum of options [2,3,4,5,6,7]. Most *Kalanchoe* germplasms were first collected from highland areas (at 1800–2000 m altitude) of Madagascar and East Africa [8]. Recent studies revealed a native species, *Kalanchoe garambiensis*, from the lowland of tropical Taiwan, and this early flowering germplasm is suitable for cultivation in tropical and subtropical regions [9,10,11]. Utilization of such wild species with valuable traits as a genetic source would be a key for reaching current breeding objectives.

Other than ornamental potted plants, the first *Kalanchoe* cut flower cultivar was launched by a Danish company, Kund Jepsen A/S, in this decade [12]. High-quality cut flowers require long stem traits and long flower longevity [13,14]. Isolation and characterization of *Kalanchoe* genetic sources for plant height, ethylene sensitivity, and longevity have been important aims for *Kalanchoe* cut flower breeding. Nevertheless, compact and dwarf plants have been classical breeding objectives of potted *Kalanchoe* for decades, and this consequently limits the genetic resources for cut flower breeding. The long stem is a common trait in section *Bryophyllum*, but the most popular *Kalanchoe* cultivars belong to the section *Kalanchoe*, and only rare cases of intersectional hybrids have been produced [4,15]. Current reports indicate the long stem trait in several species belonging to section *Kalanchoe* [15,16,17]. Using cultivars or wild species belonging to section *Kalanchoe* with this long stem trait, such as *K. nyikae* and *K. lobate*, as genetic sources is a relatively feasible option for breeding cut flower type *Kalanchoe.*

Interspecific hybridization has been an effective strategy in *Kalanchoe* breeding history [4,8,17]. Interspecific crossing between *K. blossfeldiana* and other species (e.g., *K. flammea*, *K. pumila*, and *K. marnieriana*) belonging to the same genus is often used to produce hybrids, and some hybrids are subsequently selected as new cultivars [3,8,17,18]. However, the reproductive barriers to interspecific hybridization have long been an issue for developing new *Kalanchoe* cultivars [3]. Regarding the pre-fertilization barriers, pollen-pistil interactions influence pollen germination and pollen tube growth, and several cases of these abnormalities were observed in *Kalanchoe* interspecific pollination [3]. With respect to post-fertilization barriers, the crosses between different basic ploidy or chromosome numbers are not the main factors influencing cross-compatibility, but genetic distance coinciding with the cross-compatibility [3,15,17]. Unilateral compatibility is commonly observed on *Kalanchoe* distant crosses suggesting that reciprocal crossing could be a strategy to overcome the wide cross incompatibility of certain combinations [3,4,9,15,16,18,19]. Embryo rescue has been successfully used to produce *Kalanchoe* hybrids, indicating that embryo abortion and abnormal growth are included in the reproductive barriers [18]. The occurrence of hybrid sterility results in a loss of fertility in hybrid *Kalanchoe* plants in several cases of interspecific crosses [4,17,19], and consequently limits the potential of the hybrids in further breeding programs. Chromosome doubling approaches, such as colchicine treatment, have been used to restore the fertility of sterile hybrids of ornamental plants, including *Dendrobium*, *Lilium*, *Dianthus*, and *Cyclamen* [20,21,22,23]. A fertile interspecific hybrid, *K.* × *vadensis*, has been suggested as an example to support the idea that artificial chromosome doubling can overcome hybrid sterility in *Kalanchoe* [24].

In this work, we found that the crossability of many wild accessions with long stem traits within section *Kalanchoe* are unilaterally incompatible. Among the derived F_1_ progenies, one long stemmed, large flowered and early flowered superior hybrid ‘103-1’, produced from hybridization between *K. garambiensis* and *K. nyikae,* failed to act as either a maternal or paternal parent for a further breeding program, and was therefore considered as hybrid sterile. A chromosome doubling approach revealed that the germination ability and viability of pollens from the sterile ‘103-1’ were recovered after colchicine treatment, and its fertility as both maternal and paternal parents was restored. Finally, our results reveal a successful example of restoring fertility of a novel interspecific hybrid of *K. garambiensis* and *K. nyikae* for cut flower breeding, and this hybrid further produced progeny with *K. blossfeldiana*.

## 2. Results

### 2.1. Reciprocal Crossing was Effective to Overcome Interspecific Incompatibility

To identify potential genetic resources from section *Kalanchoe* for cut flower breeding, we evaluated the crossability of collected germplasms. *K. garambiensis* ‘Type 1’ and *K. garambiensis* ‘Type 2’ were two clones selected from endemic *K. garambiensis* Kudo populations collected from lowland areas of tropical Taiwan. This tropical wild species is an early flowering plant that requires less short-day exposure for flower initiation. Other than genetic sources for suitable photoperiodism and adaptation, *K. nyikae*, *K. lobata*, *K. velutina*, *K. sexangularis*, *K. longiflora*, and *K. blossfeldiana* ‘African Love’ were wild species, and a cultivar, with long stem traits. Among the tested hybridization pairs, the wide cross incompatibilities of most pairs were unilaterally incompatible and could be overcome by reciprocal crossing, except that some pairs were compatible and that crosses between *K. lobata* and *K. nyikae* were incompatible (Table 1). This unilateral incompatibility was observed from two aspects: seed production and seed germination. Regarding the crosses between *K. garambiensis* ‘Type 1’ and *K. nyikae*, using *K. garambiensis* ‘Type 1’ as the maternal parent produced 110.8 seeds per capsule, while when using *K. nyikae* as the maternal parent only 1.2 seeds per capsule were harvested (Table 1), suggesting that the unilateral incompatibility between *K. garambiensis* ‘Type 1’ and *K. nyikae* occurred from pre-fertilization to seed development. Another reciprocal cross between *K. garambiensis* ‘Type 2’ and *K. velutina* produced only 1.3 seeds per capsule when *K. velutina* was the maternal parent (Table 1). Notably, the seeds from this low-producing cross had higher germination (57.1%), while seeds from the normal-producing cross (141 seeds per capsule) showed low germination (2.8%) (Table 1). These results suggest that unilateral incompatibility is caused by several decouplable causes, such as pre-fertilization barriers, seed development, and seed germination. Among the tested crosses, except for the combination of *K. lobata* and *K. nyikae,* which failed to obtain a hybrid, reciprocal crossing could overcome wide cross incompatibility of interspecific crosses within section *Kalanchoe*.

### 2.2. Selfing and Backcrossing Are not Sufficient to Rescue the Sterility of F_1_ from Interspecific Crosses of K. garambiensis ‘Type 1’ × K. nyikae

**Figure 1:**
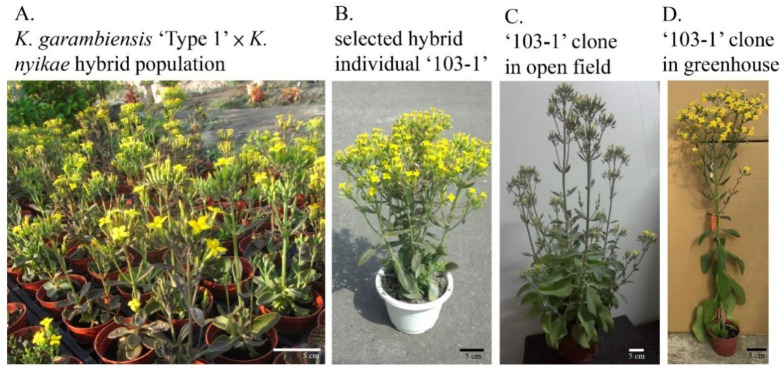
Appearance of hybrid ‘103-1’ derived from *K. garambiensis* ‘Type 1’ × *K. nyikae*. (**A**) Part of the hybrid population of *K. garambiensis* ‘Type 1’ × *K. nyikae,* (**B**) The ‘103-1’ hybrid individual selected from the hybrid population of *K. garambiensis* ‘Type 1’ × *K. nyikae* cultivated on an open field. (**C**) A clone of ‘103-1’ propagated from a shoot tip, and cultivated on an open field. (**D**) A clone of ‘103-1’ propagated from a shoot tip and cultivated in a shaded greenhouse.

Among the derived hybrids, a superior hybrid ‘103-1’ was selected form the hybrid population (Figure 1A) of *K. garambiensis* ‘Type 1’ × *K. nyikae*, which developed large florets and long stems (Figure 1B). Clonally propagated ‘103-1’ reproduced similar long stem traits (Figure 1C) to the selected individual, and less branching and a longer stem were observed when ‘103-1’ was cultivated in a shaded green house (Figure 1D), indicating that shading could result in the generation of suitable traits for cut flowers in ‘103-1’.

Given the valuable cut flower traits of ‘103-1’, this clone was considered as a potential parent for our further breeding program. However, no seeds were obtained for any executed crosses. Since backcross and selfing have been potential strategies to produce seed from hybrids [25], these were carried out in ‘103-1’ (Table 2). Neither of these methods resulted in any seeds. Another fertile hybrid ‘103-2’ selected from the *K. spathulata* × *K. garambiensis* ‘Type 1’ population was able to produce seeds by selfing or crossing with *K. nyikae* and *K. blossfeldiana* ‘Cher’ (Table 2). These results support the idea that ‘103-1’ is hybrid sterile.

### 2.3. Colchicine Treatment on Apical Buds of ‘103-1’ Successfully Produce Viable Pollen with Normal Releasing, Morphology and Germination

**Figure 2:**
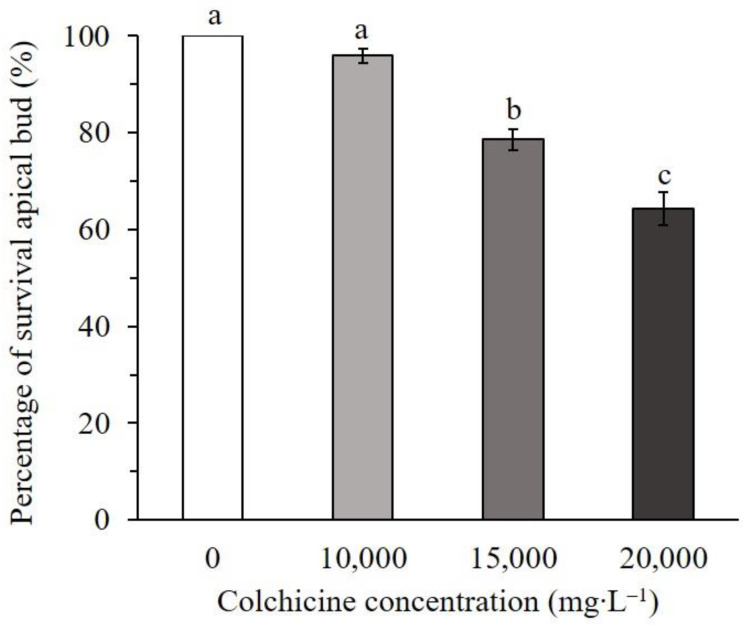
Effect on percentage survival of apical buds in ‘103-1’ (*Kalanchoe garambiensis* ‘Type 1’ × *K. nyikae*) at different concentration of colchicine treatment. The data represent means ± SD from three repeats (n = 32 to 33 plants). Different letters indicate significant differences between each other at *p* < 0.05 using one-way ANOVA with Tukey’s HSD test.

**Figure 3:**
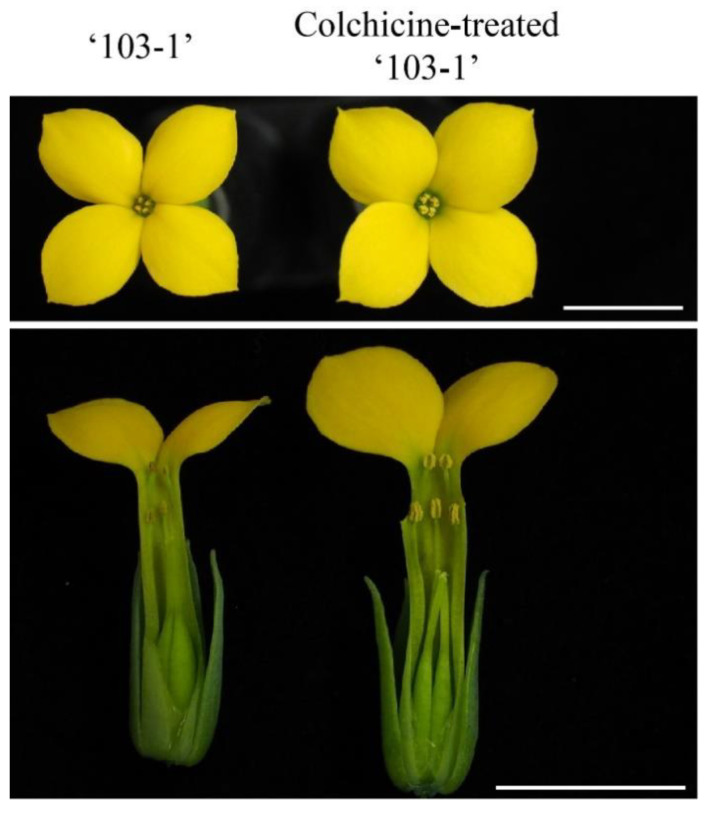
Pollen release from anthers of ‘103-1’ (*Kalanchoe garambiensis* ‘Type 1’× *K. nyikae*) was rescued by colchicine treatment. Control (**left**) shows abnormal pollen generation and 10,000 mg∙L^−1^ colchicine-treated plant (**right**) shows normal pollen release from the anther. The upper panel shows top views of flowers, the bottom panel shows side views of flowers. Scale bar = 10 mm.

**Figure 4:**
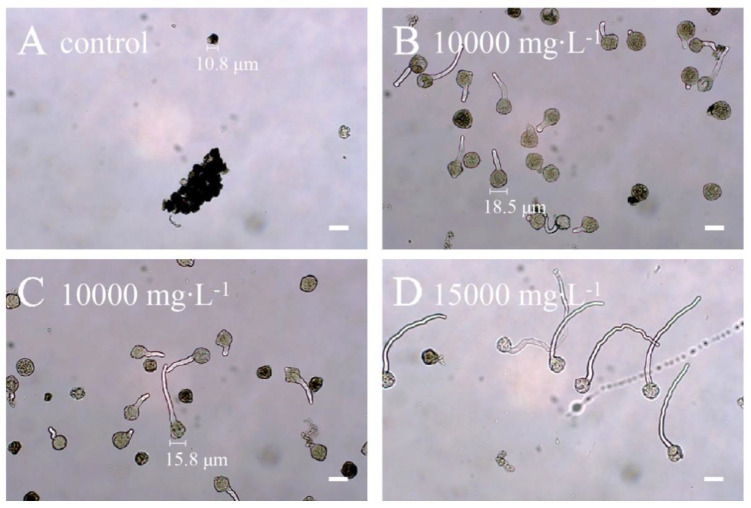
Abnormalities in pollen morphology and germination from ‘103-1’ (*Kalanchoe garambiensis* ‘Type 1’ × *K. nyikae*) were restored in colchicine-treated ’103-1’ clones. (**A**) No colchicine control ‘103-1’ clone generated pollens with abnormal morphology and no germination observed. (**B**,**C**) are from two individual 10,000 mg∙L^−1^ colchicine-treated ’103-1’ clones. Normal morphology and pollen germination were observed with germination percentages of 17.3% and 21.3%, respectively. (**D**) is from a 15,000 mg∙L^−1^ colchicine-treated ’103-1’ clone with pollen germination percentage at 86.7%. Bar = 20 μm. Sizes of selected pollen grains were labelled in each panel.

**Figure 5:**
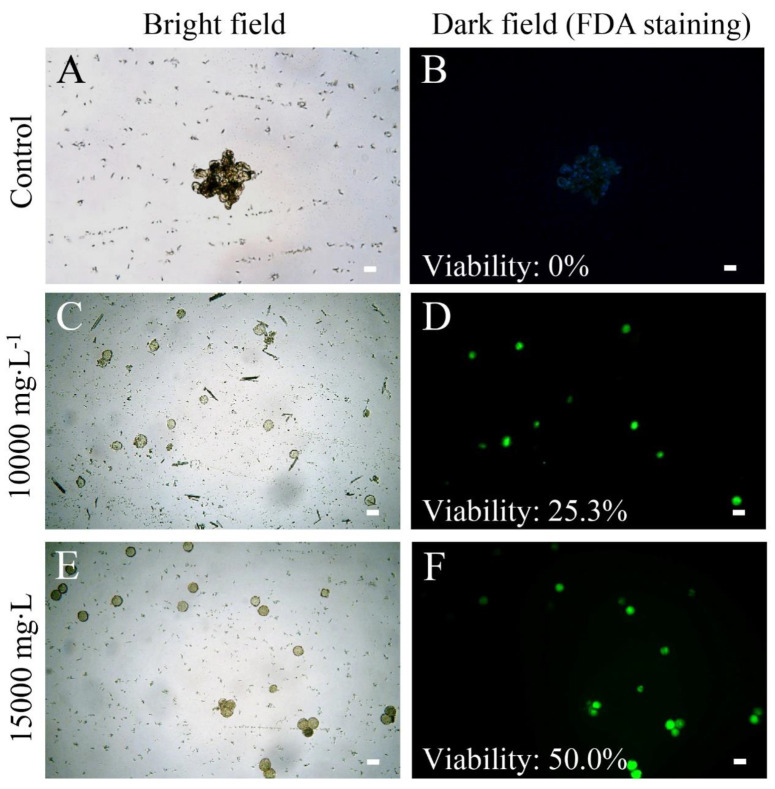
Pollen viability of ‘103-1’ (*Kalanchoe garambiensis* ‘Type 1’ × *K. nyikae*) was restored in colchicine-treated ’103-1’ clones using fluorescein diacetate (FDA) viability staining. (**A**,**B**), no colchicine control ‘103-1’ clone produced aggregated pollen with no observable viability. (**C**,**D**), a 10,000 mg∙L^−1^ colchicine-treated ‘103-1’ clone produced pollens with 25.3% of viability. (**E**,**F**), a 15,000 mg∙L^−1^ colchicine-treated ‘103-1’ clone produced pollens with 50.0% of viability. Scale bar = 20 μm.

**Figure 6:**
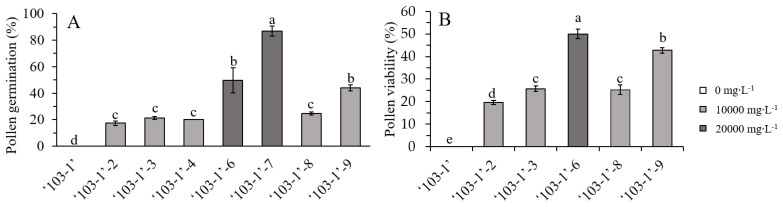
Percentage of pollen germination (**A**) and viability (**B**) in ‘103-1’ (*Kalanchoe garambiensis* ‘Type 1’ × *K. nyikae*) and colchicine-treated ‘103-1’ clones. The data represent means ± SD from three repeats (n > 100 pollens). Different letters indicate significant differences between each other at *p* < 0.05 using one-way ANOVA with Tukey’s HSD test.

To restore fertility of ‘103-1’, apical buds were treated with 0, 10,000, 15,000, and 20,000 mg∙L^−1^ of colchicine in lanolin for chromosome doubling. The toxicity of colchicine would lead to damage of apical buds, and a higher concentration of colchicine reduced the survival of apical buds, but surviving buds were observed in all treated concentrations (Figure 2). Pollen release from the anthers was easily observed on the flowers of colchicine-treated ‘103-1’, whereas shrunk anthers and no obvious pollen release was observed in non-treated ‘103-1’ plants (Figure 3). To further assess pollen fertility, pollen germination testing and pollen viability staining were carried out. Aggregated and shrunk pollens were found in the anther of ‘103-1’, and these abnormal pollens failed to germinate (Figure 4A). The pollen collected from three individual colchicine-treated ‘103-1’ plants showed normal pollen morphology and was able to germinate (Figure 4B–D). Pollen viability was evaluated using fluorescein diacetate (FDA) staining under fluorescence microscopy. The aggregated pollens collected from ‘103-1’ without colchicine treatment showed no viability by FDA staining (Figure 5A,B). Pollens produced from colchicine-treated ‘103-1’ are viable (Figure 5C–F). Among colchicine-treated ‘103-1’ clones, eight clones were selected to develop flowers and evaluate pollen germination and viability (Figure 6). Pollens from all eight clones were able to germinate, with germination percentages ranging from 17.3% to 86.7% (Figure 6A). With regard to pollen viability, among the five assessed clones, all five had viable pollen with viability percentages ranging from 19.7% to 50% (Figure 6B). These results indicate that colchicine treatment on apical buds at a concentration of 10,000 and 15,000 mg∙L^−1^ in lanolin is effective at restoring pollen germination and pollen viability in the ‘103-1’ hybrid.

### 2.4. Proportion of Doubled Ploidy Nuclei Increases in Fertile Colchicine-Treated ‘103-1’

**Figure 7:**
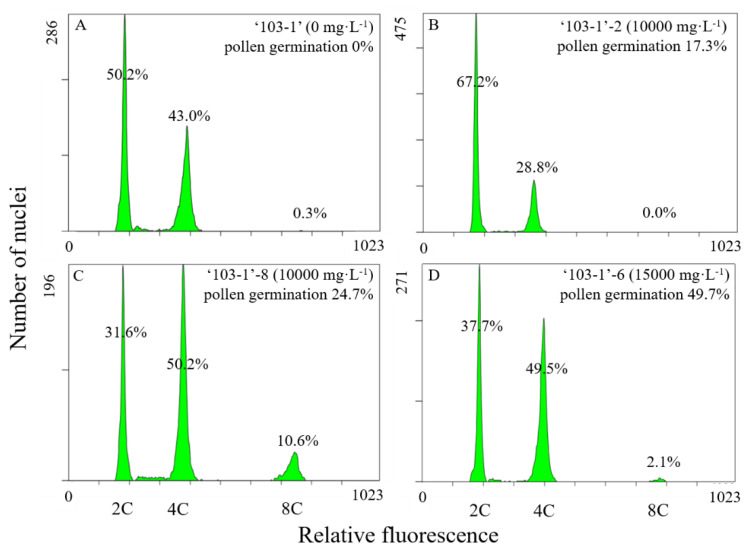
Flow cytometric patterns of leaves obtained from ‘103-1’ hybrid (*Kalanchoe garambiensis* ‘Type 1’ × *K. nyikae*) and colchicine-treated ‘103-1’ clones. (**A**) Control, ‘103-1’ treated with lanolin only without colchicine. (**B**,**C**) are ‘103-1’-2 and ‘103-1’-8, respectively. Both clones were obtained from ‘103-1’ after treatment with 10,000 mg∙L^−1^ colchicine in lanolin. (**D**) shows ‘103-1’-6, which were obtained from ‘103-1’ after treated with 15,000 mg∙L^−1^ colchicine in lanolin.

Restored fertility of pollen from colchicine-treated ‘103-1’ clones was expected as an outcome of induced chromosome doubling. Flow cytometric analysis has been an efficient and popular method to determine ploidy levels for higher plants, based on the precise measurement for DNA content of individual nuclei from plants with different ploidy levels [26,27,28]. To evaluate the ploidy level of these colchicine-treated ‘103-1’ clones, flow cytometric analysis was conducted to profile DNA content of nuclei isolated from leaves of these clones (Figure 7). Control ‘103-1’ had two peaks. The major nuclei count (50.2%) peak with weaker fluorescence showed the DNA content of G1 cells that was considered as the diploid 2C peak, whereas another peak with double DNA fluorescence indicated G2 cells and cells of polysomaty that were recognized as the tetraploid 4C peak (Figure 7A). Three colchicine-treated ‘103-1’ clones, ‘103-1’-2, ‘103-1’-8, and ‘103-1’-6, were subjected for flow cytometric analysis (Figure 7B–D). Two out of the three clones, ‘103-1’-8, and ‘103-1’-6, showed that the 4C peak became the major nuclei count peaks, and 8C peaks were also observed (Figure 7C,D). Coincidentally, ‘103-1’-2, a colchicine-treated ‘103-1’ clone with the lowest pollen germination among tested colchicine-treated clones (Figure 6A), did not show increases in the 4C and 8C peaks (Figure 7B), suggesting that the minor restoration of pollen fertility may be led by a very low proportion of doubled ploidy cells in the mixoploids clone, and this low proportion was too low to be detected. Taken together, the results suggest that the fertile colchicine-treated ‘103-1’ clones were mixoploids, and higher proportions of doubled ploidy were observed in these clones.

### 2.5. Colchicine-Treated ‘103-1’ Restores Maternal Fertiltiy to Bear Vaiable Seeds

To further evaluate the maternal fertility of these colchicine-treated ‘103-1’ clones that have restored pollen fertility, several colchicine-treated ‘103-1’ clones were open pollinated, and their ability to bear seeds and the germination of these seeds were then assessed (Table 3). Among the ten assessed clones, seeds could be harvested from five colchicine-treated ‘103-1’ clones. Germination percentage of the seeds was higher than 50% for each clone, and their seedlings developed normally. Regarding the other five clones that failed to bear seeds, two were treated with 15,000 mg∙L^−1^ colchicine, and the other three clones were treated with 20,000 mg∙L^−1^ colchicine. Notably, four out of five maternal fertile clones were treated with 10,000 mg∙L^−1^ colchicine, suggesting an optimal colchicine treatment condition. These results supported that colchicine treatments not only restore the pollen fertility of sterile ‘103-1’ hybrid, but also facilitate recovery of maternal fertility.

### 2.6. Colchicine-Treated ‘103-1’ Could Be Pollen Donor for Further Hybridization Breeding Program

To utilize the fertility restored ‘103-1’ clones for further breeding program, it is necessary to assess the crossability of the clones to other germplasms. The pollen collected from fertile ‘103-1’-1 and ‘103-1’-3 were used to fertilized *K. nyikae* and *K. blossfeldiana* ‘Ida’, respectively (Table 4). Although seed number was relatively low per capsule in both crosses, both hybridization combinations are capable of producing seeds with a high germination percentage (88.9% to 100%), and progenies of these hybridizations were obtained.

## 3. Discussion

Here, we report novel combinations of interspecific hybridization for breeding cut flowers of *Kalanchoe*. We demonstrated that reciprocal cross is a recommended strategy for overcoming unilateral incompatibility, a frequent occurrence of a wide cross barrier in *Kalanchoe* interspecific hybridization. Hybrid sterility is another obstacle, even when superior hybrid individuals are selected. Sterility limits the use of the derived hybrids for further breeding programs. Using artificial chromosome doubling by colchicine treatment, we were able to demonstrate an example of fertility restoration for a sterile superior hybrid ‘103-1’. This fertility restoration reveals many aspects supporting recovery from sterility, including pollen germination, pollen viability, maternal fertility, seed germination, and ploidy. Via crossing the fertility-restored hybrid ‘103-1’ clones with other germplasms, we then demonstrated that the restored clones can be utilized in further cut flower breeding programs.

The classical breeding objectives of *Kalanchoe* cultivars focus on flower color, double flowers, and compactness [8]. *Kalanchoe* breeding began in the 1920s, when the first *K. blossfeldiana* was introduced to Europe, followed by interspecific hybridization with *K. flammea* in the late 1930s [8]. After decades of breeding with a narrow target trait focus, the genetic variations are mainly restricted in *K. blossfeldiana*-derived cultivars. Moreover, dwarfness and compactness are traits that are suitable for potted *Kalanchoe* breeding, but not for cut flower breeding. Introduction of wild species with long stems has recently been considered a potential approach for cut flower breeding [16]. Most species belonging to section *Bryophyllum* are long stems, but only a few cases of intersectional hybrids between section *Bryophyllum* and section *Kalanchoe* was successful, and the derived intersectional hybrid failed to bear seeds [4,15,16]. The present study collected several wild species and cultivars belonging to the section *Kalanchoe*, which has the long stem trait [15,16,17]. Ten out of eleven intra-sectional hybridization pairs within the section *Kalanchoe* successfully produced viable seeds (Table 1). Even though one derived superior hybrid is sterile, this work takes advantage of chromosome doubling to restore the fertility of the hybrid. Intra-sectional hybridization is likely to be a relatively feasible approach to introduce new traits and to increase genetic variability.

Photoperiodism, days to flowering, and environmental adaptation are important traits for the production of cut flowers and potted flowers. A current report characterized flower development and photoperiodism for two Taiwan endemic species, *K. garambiensis* and *K. spathulate*, revealing that *K. garambiensis* is a free-branching and mid-flowering plant, and *K. spathulate* is an early flowering species [10]. Other than the earlier flowering time, the distribution of these two wild species is located at low altitudes in tropical and subtropical areas, whereas most current cultivars are derived from species collected at an altitude of 1800–2000 m in Madagascar and East Africa [8]. Origins of *K. garambiensis* and *K. spathulate* suggest that their derivatives could carry genetic variability for adapting tropical and subtropical cultivation in low land areas. Although the adaptation of ‘103-1’ in tropical and subtropical regions has not yet been well characterized, this *K. garambiensis* derived hybrid flowered earlier than other *K. blossfeldiana* cultivars, and has the potential to adapt to tropical and subtropical regions based on our preliminary observations. Shading could be another condition to improve cut flower quality of ‘103-1’. Longer stem and branch-free ‘103-1’ plants were observed in shaded (around 40% to 60% light intensity of open field) greenhouse cultivation (Figure 1C,D). Further investigation into adaptation and shading effects could be an important issue for future breeding programs.

Reproductive barriers hamper the breeding of interspecific hybrids for *Kalanchoe*, which are typically recognized as pre- and post-fertilization barriers [3]. Pre-fertilization barriers are often associated with pollen germination and the growth of pollen tubes on the stigma [3,9]. When *K. garambiensis* is the pollen donor to cross *K. blossfeldiana* ’Isabella’, pollens are poorly germinated with abnormal and arrested pollen tubes, but normal pollen germination and pollen tube penetration into ovules occurs following reciprocal pollination using *K. blossfeldiana* ’Isabella’ as the pollen donor [9]. This case of a pre-fertilization barrier could be a reason to explain unilateral incompatibility in *Kalanchoe*. The interspecific cross between *K. garambiensis* ‘Type 1’ and *K. nyikae* shows similar incompatibility, in which lots of seeds produced when *K. garambiensis* ‘Type 1’ is maternal parent but very few seeds are produced when *K. garambiensis* ‘Type 1’ is the pollen donor (Table 1), suggesting that the unilateral incompatibility in our study could be caused by similar pre-fertilization mechanisms.

Low production and germination of seeds in hybridization may also be triggered by post-fertilization barriers. Ovule culture has been successfully rescued using hybrid embryos in the crosses of *K. blossfeldiana* with *K. daigremontiana*, *K. laxiflora*, *K. citrina*, *K. garambiensis*, *K. pumila* and *K. spathulata* [18]. This report supports the idea that incompatibility could be associated with embryo development and subsequently affects seed production and germination. In the current study, low germination (e.g., zero germination out of 243 seeds in the cross of *K. sexangularis* × *K. longiflora*) was observed (Table 1), which could be attributed to abnormal embryo development or hybrid unviability. Hybrid sterility is another common type of post-fertilization barrier. The progeny of *K. garambiensis* ‘Type 1’ × *K. nyikae* was successfully produced in this work and considered as a potential genetic resource for further cut flower breeding; however, this hybrid is sterile. Similar sterility has been reported when pollens from hybridization between *K. manginii* ‘Wendy’ and *K. blossfeldiana* ‘Isabella’ failed to germinate [19]. Chromosome doubling has been used to restore the fertility of sterile hybrids in other ornamental plants [20,21,22,23]. In the *Kalanchoe* genus, a fertile interspecific hybrid, *K.* × *vadensis* developed in the 1960s has been considered as an example of a fertility-restored hybrid using artificial chromosome doubling [24]. Spontaneous chromosome doubling in leaf tissue culture has successfully doubled the ploidy of sterile hybrids from the cross of *K. blossfeldiana* with *K. daigremontiana*, *K. laxiflora*, and *K. farinacea*, but the restoration of their fertility has not yet been reported [18]. In the current study, artificial chromosome doubling by colchicine was used to produce fertile mixoploid hybrids in a novel cross between *K. garambiensis* ‘Type 1’ and *K. nyikae*. Nevertheless, a recent case of intersectional hybrids derived from *K. blossfeldiana* ‘Hayworth’ × *K. pinnata* failed to restore fertility by chromosome doubling using colchicine-treated shoot tissue culture [16]. Taken together, artificial or spontaneous chromosome doubling could be useful techniques to restore fertility in some cases of sterile hybrids from wide crosses.

Chromosome structure, chromosome number, and ploidy level are often recognized as mechanisms causing intrinsic hybrid unviability and sterility [29]. Among species in this study with known chromosome information, all species have identical basic chromosome number (x = 17), but some species are diploids (i.e., *K. velutina* Welw., *K. sexangularis* N. E. Brown and *K. longiflora* Schltr. (2n = 2× = 34)), and *K. garambiensis* Kudo, *K. nyikae* Engler, and *K. blossfeldiana* von Poelln. cultivars are tetraploids (2n = 4× = 68, see Table 5). Notably, reciprocal effects on seed production were observed in the interploidy crosses (Table 1). Low seed production (0.5 to 4 seeds per capsule) occurred in the interploidy crosses when *K. velutina* (2n = 34) was used as maternal parent and *K. garambiensis* ‘Type 1’ (2n = 68), *K. garambiensis* ‘Type 2’ (2n = 68) or *K. nyikae* (2n = 68) was used as paternal parent. On the contrary, higher seed production was observed when higher ploidy level was maternal parent (2n = 68) to cross with lower ploidy level (2n = 34), in which interploidy crosses were *K. garambiensis* ‘Type 2’ (2n = 68) × *K. velutina* (2n = 34) produced 141 seeds per capsule, *K. nyikae* (2n = 68) × *K. velutina* (2n = 34) produced 178.7 seeds per capsule, and *K. nyikae* (2n = 68) × *K. sexangularis* (2n = 34) produced 494 seeds per capsule. Although we cannot exclude the possibility that *K. velutina* is a low-seed-production maternal parent, this result suggested the involvement of incompatible endosperm balance number (EBN) in *Kalanchoe* interspecific barriers. The EBN model is based on the nature of the endosperm that is contributed by two maternal and one paternal genomes, and the unbalanced parental genome dosage would cause endosperm failure and consequently lead to embryo arrest [30,31,32]. Similar cases of interploidy crosses have been also reported, e.g., *K. blossfeldiana* ‘0089A’ (2n = 68) × *K. pubescens* (2n = 34) produced 2.9 seeds per capsule, but *K. pubescens* × *K. blossfeldiana* ‘0089A is sterile [3]. These consistent cases support the hypothesis that endosperm development in *Kalanchoe* interploidy hybridized seeds is sensitive to lower maternal dosage. Even though a recent report states that genetic distance is a major factor related to cross-compatibility of *Kalanchoe,* and differences in ploidy is a minor factor when influencing the success of interspecific crosses [3], considering that further hybridization of the chromosome-doubled fertile ‘103-1’ with other cultivars is likely to be interploidy hybridization, EBN is still a potential issue in the future breeding program.

Various application methods and doses of colchicine treatments have been conducted for chromosome doubling in other ornamentals. Cyclamen hybrid ovules treated in vitro with 0.05% colchicine in MS medium for 10 to 15 days can produce fertile plants [23]. Sterile hybrid obtained from an interspecific hybridization between carnation (*Dianthus caryophllus*) and *Dianthus japonicus* is restored to fertile amphidiploid by either applying drops of 2000 mg∙L^−1^ colchicine on shoot tips or in vitro shaking culture of nodal segments with 5 to 10 mg∙L^−1^ colchicine for 1 day [21]. A chromosome doubling protocol was developed to restore the fertility of F_1_ interspecific hybrid in *Lilium*. This protocol utilized bulblets pre-cultured from basal scale segments followed by soaking with 1.25 mM colchicine (equivalent ~499.3 mg∙L^−1^) for 24 h [22]. In our study, 10,000 mg∙L^−1^ colchicine-lanolin paste applied on the primary shoot apex was effective in inducing chromosome doubling for *Kalanchoe*. By comparing colchicine concentrations used in other ornamentals (i.e., ranging from 5 to 500 mg∙L^−1^ in tissue culture, and 2000 mg∙L^−1^ in dropping on shoot tip), 10,000 mg∙L^−1^ colchicine is still relatively high. Since over-dosage of colchicine would be harmful for plants, optimizing colchicine dose with concentrations lower than 10,000 mg∙L^−1^ could be potential options. Instead of treating in vitro cultures, our study contributes a convenient protocol by directly treating shoot apex with colchicine paste for ornamental chromosome doubling.

In conclusion, the introduction of a wild species originating from lowland tropical regions and a collection of long stem germplasms highlights a promising gene pool for *Kalanchoe* cut flower breeding. Although novel marketable cultivars have yet to be developed, this study provides an idea for feasible breeding programs that adopt strategies to overcome the reproductive barriers and produce hybrid populations and fertile hybrid individuals for further breeding. Understanding the mechanisms that lead to reproductive barriers is the key to developing new hybrid cultivars. The next challenge is to further cross fertile hybrids developed in this study, followed by selection and characterization of superior hybrid progeny.

## 4. Materials and Methods

### 4.1. Plant Materials

Ten genotypes belonging to seven *Kalanchoe* species were used in this study: two selected clones of Taiwan endemic species *K. garambiensis* Kudo ‘Type 1’ and ‘Type 2’, three cultivars of *K. blossfeldiana* von Poelln. ‘African Love’, ‘Cher’, and ‘Ida’, and five long stem wild species: *K. nyikae* Engler, *K. lobata* R. Fern., *K. velutina* Welw., *K. sexangularis* N. E. Brown, and *K. longiflora* Schltr. All *Kalanchoe* germplasms were from Yi Kuang’s private collection. The selected hybrid individuals, ‘103-1’ and ‘103-2’, were provided by a private breeder collaborator, Cheng-Chieh Hsieh, who generates *K. garambiensis* ‘Type 1’ × *K. nyikae* hybrid population and selected ‘103-1’. The selection of ‘103-1’ (Figure 1A,B) and open-field cultivation of ‘103-1’ clone were carried out in Cheng-Chieh Hsieh’s private farm in Tianwei Township, Changhua County, Taiwan. The plants for the experiments were established by cutting shoot tips with two pairs of leaves in 2-inch plastic pots with commercial medium (Potgrond H, Klasmann-Deilmann GmbH, Geeste, Germany). The plants were watered twice per week with tap water and cultivated in greenhouse until roots reached plastic pots. After one to two months, the developed plants were transferred to three-inch pots with a mixture of two volumes of peat moss (Kekkilä Estonia Peat Moss, Kekkilä Oy, Vantaa, Finland) and one volume of perlite. The plants were watered twice per week with a solution containing 0.5 g∙L^−1^ soluble fertilizer (Peters 20-20-20, The Scotts Miracle-Gro Company, Marysville, OH, USA). The plants were cultivated in the greenhouse of Experimental Farm (25°00′43.7″ N 121°32′49.7″ E), College of Bioresources and Agriculture at National Taiwan University under natural photoperiod.

The first step of pollination and crossing was emasculation, which was carried out one day before flower opening. Both anthers and petals were removed. Pollination was conducted two to three days after emasculation until mucus was secreted on stigma by brushing fresh pollens. Capsules were collected 40 days to 3 months after pollination when capsules were dried. Only filled seeds were counted. For assessing germination percentage, at least 30 seeds and three repetitions were carried out on three-inch pots with commercial medium (Potgrond H, Klasmann-Deilmann GmbH, Geeste, Germany).

### 4.2. Colchicine Treatment

The required amount of colchicine (Alfa Aesar, Ward Hill, MA, USA) was dissolved in melted lanolin. Three colchicine concentrations were prepared: 10,000, 15,000, and 20,000 mg∙L^−1^, equivalent to 10.57, 15.86, and 21.15 mg∙g^−1^, respectively. The colchicine-lanolin paste was applied on the primary shoot apex on 23 August 2017, and the application was repeated one week after the first treatment. The survival of apical buds was recorded on 30 September 2017 with 33 to 34 plants in each treatment with three replicates.

### 4.3. In Vitro Pollen Germination

Pollens for assessing germinability was collected from flowers on the first day of flower opening for each individual plant. Flowering time was from February to March, and in vitro pollen germination was carried out from February to March in 2017 and 2018. The in vitro pollen germinability was assessed in liquid medium, as previously described, with minor modifications [36]. The medium contained 200 mg∙L^−1^ MgSO_4_, 300 mg∙L^−1^ Ca(NO_3_)_2_, 100 mg∙L^−1^ KNO_3_, 100 mg∙L^−1^ H_3_BO_3_, and 5% (*w*/*v*) sucrose at pH 6.0. Each repeat collected pollen from an individual flower was transferred into a 1.5-mL tube with 1 mL of the liquid medium. After gentle agitation, the tubes were incubated in a 25 °C water bath in the dark for 2 h. The incubated pollen grains were analyzed using a light microscope (Leica DMLS Microscope, Leica, Wetzlar, Germany). The microscope images were captured using a CMOS microscope camera (Mshot MD50, Micro-shot Technology, Guangzhou, China). At least 100 pollen grains were observed for each treatment with three replicates.

### 4.4. Pollen Viability

Pollen for assessing viability was collected from flowers on the first day of flower opening. Pollens from a single flower was resuspended in 1 mL of pollen germination medium, as mentioned previously (Materials and Methods Section 4.3). For viability staining, 0.2 μL of 2 mg mL^−1^ fluorescein diacetate (Sigma-Aldrich, St. Louis, MO, USA) stock in acetone was added to the 1 mL pollen suspension. The stained pollen grains were observed using a light microscope (Leica DMLS Microscope, Leica, Wetzlar, Germany) for the bright-field images. The pollen viability fluorescence was analyzed using an LED fluorescence illuminator (MF-BG(U)-LED, Micro-shot Technology, Guangzhou, China) that was adopted on the light microscope. The dark field was under excitation filter wavelength of 460–490 nm and emission barrier filter wavelength >510 nm. Images were acquired using a CMOS microscope camera (Mshot MD50, Micro-shot Technology, Guangzhou, China). The pollen grains were scored: green fluorescence as viable and unstained as unviable. At least 100 pollen grains were observed for each treatment with three replicates.

### 4.5. Determination of Ploidy Using Flow Cytometric Analysis

The first to third pairs of fully developed leaves were analyzed by flow cytometry, as previously described with minor modifications [28]. Briefly, approximately 1 g of fresh leaf tissues was placed in a Petri dish containing 1 mL of ice-cold Galbraith’s buffer (45 mM MgCl_2_, 20 mM MOPS, 30 mM sodium citrate, 0.1% (*v*/*v*) Triton X-100, pH 7.0, adjusted with NaOH). The leaf tissues were immediately chopped with a new razor blade in buffer. The homogenate was then mixed by pipetting, and filtered through a presoaked 40-μm nylon mesh (Falcon 40 µm Cell Strainer, Corning, Corning, NY, USA), followed by incubation at 4 °C for 5 min. The presence of nuclei and free of other particles in the filtrate were checked visually under a light microscope (Leica DMLS Microscope, Leica, Wetzlar, Germany) before conducting flow cytometric analysis. To stain nuclear DNA, the filtrate was centrifuged at 60× *g* and 4 °C for 3 min to remove the supernatant, followed by the addition of 100 μL of ice-cold Galbraith’s buffer and 150 μL of 1 mg mL^−1^ propidium iodide (Sigma-Aldrich, St. Louis, MO, USA). After staining with propidium iodide on ice for 10 min in the dark, flow cytometric analysis was conducted using a flow cytometer (FC500, Beckman Coulter, Brea, CA, USA). At least 1000 nuclei were analyzed for each sample.

### 4.6. Statistical Analysis

The significance differences was conducted with two methods. For comparing more than three groups, one-way analysis of variance (ANOVA) was employed followed post-hoc analysis by Tukey’s honestly significant difference test (HSD). For comparing two groups, Student’s *t* test was carried out. All analysis was conducted using the statistical package, SPSS version 22 (SPSS, Chicago, IL, USA).

## Figures and Tables

**Table 1:** Reciprocal crossing could overcome wide cross incompatibility of interspecific crosses within the section *Kalanchoe*.

Maternal Donor	Pollen Donor	Mean (Seed Number/Capsule, SD)	Seedling Number ^1^	Germination Percentage (SD) ^2^
*K. garambiensis* ‘Type 1’	*K. nyikae*	110.8 (997/9, 75.6) ^c^	229	23 (5.6) ^efg^
*K. nyikae*	*K. garambiensis* ‘Type 1’	1.2 (6/5, 0) ^d^	0	0 ^‡^
*K. garambiensis* ‘Type 1’	*K. lobata*	322.5 (1290/4, 80.8) ^a^	937	72.6 (8.9) ^ab^
*K. lobata*	*K. garambiensis* ‘Type 1’	91 (182/2) ^†^	112	61.5 (7.5) ^bc^
*K. velutina*	*K. garambiensis* ‘Type 1’	4 (28/7, 0.5) ^d^	15	53.6 ^‡^
*K. garambiensis* ‘Type 2’	*K. lobata*	133.3 (400/3, 34.0) ^c^	75	18.8 (7.2) ^fgh^
*K. lobata*	*K. garambiensis* ‘Type 2’	154 (154/1) ^†^	84	54.5 (6.5) ^bcd^
*K. garambiensis* ‘Type 2’	*K. velutina*	141 (141/1) ^†^	4	2.8 (1.0) ^gh^
*K. velutina*	*K. garambiensis* ‘Type 2’	1.3 (14/11, 0.5) ^d^	8	57.1 ^‡^
*K. lobata*	*K. velutina*	204 (204/1) ^†^	84	41.2 (7.3) ^cde^
*K. velutina*	*K. lobata*	0 (0/1) ^†^	- ^3^	-
*K. lobata*	*K. nyikae*	17 (119/7, 43.0) ^d^	0	0 ^h^
*K. nyikae*	*K. lobata*	0 (0/2) ^†^	-	-
*K. velutina*	*K. nyikae*	0.2 (3/14, 0.3) ^d^	1	33.3 ^‡^
*K. nyikae*	*K. velutina*	178.7 (536/3, 11.5) ^b^	474	88.4 (3.8) ^a^
*K. sexangularis*	*K. longiflora*	81 (243/3, 17.5) ^c^	0	0 (0.0) ^a^
*K. nyikae*	*K. sexangularis*	494 (494/1) ^†^	188	38.1 (7.7) ^def^
*K. blossfeldiana* ‘African Love’	*K. lobata*	24.3 (730/30, 5.0) ^d^	488	66.8 (8.1) ^ab^
*K. lobata*	*K. blossfeldiana* ‘African Love’	15.3 (107/7, 5.7) ^d^	64	59.8 (8.1) ^bc^

^1^ Seedling number: total seedlings obtained from all collected seeds. ^2^ Germination percentage: at least 30 seeds and 3 repetitions were carried out. ^3^ -, indicates not applicable. Different letters indicate significant differences between each other at *p* < 0.05 (one-way ANOVA with Tukey’s honestly significant difference (HSD) test). ^†^ indicates not applicable for statistical analysis (capsule number <3). ^‡^ total seed number <90, not applicable for statistical analysis because seeds were not enough for three replicates with at least 30 seeds in each germination test. SD, standard deviation.

**Table 2:** Selfing and backcrossing are not sufficient to rescue the sterility of F_1_ from interspecific crosses of *K. garambiensis* ‘Type 1’ × *K. nyikae*.

Maternal Donor	Pollen Donor	Mean (Seed Number /Capsule, SD) ^3^	Seedling Number	Germination Percentage (SD) ^4^
‘103-1’ ^1^	‘103-1’	0 (0/28, 0.0) ^b^	- ^5^	-
‘103-1’	*K. garambiensis* ‘Type 1’	0 (0/85, 0.0) ^b^	-	-
‘103-1’	*K. nyikae*	0 (0/63, 0.0) ^b^	-	-
‘103-2’ ^2^	‘103-2’	35 (35/1) ^†^	13	37.1 ^‡^
‘103-2’	*K. nyikae*	287.8 (1151/4, 79.2) ^a^	84	7.3 (4.6) ^b^
*K. nyikae*	‘103-2’	36 (72/2) ^†^	0	0 ^‡^
‘103-2’	*K. blossfeldiana* ‘Cher’	12.3 (98/8, 6.7) ^b^	24	24.5 (4.7) ^a^
*K. blossfeldiana* ‘Cher’	‘103-2’	2.2 (38/17, 3.8) ^b^	22	57.9 ^‡^

^1^ ‘103-1’ (*K. garambiensis* ‘Type 1’ × *K. nyikae*). ^2^ ‘103-2’ (*K. spathulata* × *K. garambiensis* ‘Type 1’). ^3^ Seedling number: total seedlings obtained from all collected seeds. ^4^ Germination percentage: at least 30 seeds and 3 repetitions were carried out. If seeds are not enough for repetitions, all collected seeds were germinated. ^5^ -, indicates not applicable. Different letters indicate significant differences between each other at *p* < 0.05 using one-way ANOVA with Tukey’s HSD test for seed number/capsule, or at *p* < 0.001 using Student’s *t* test for germination percentage. ^†^ indicates not applicable for statistical analysis (capsule number <3). ^‡^ total seed number <90, not applicable for statistical analysis because seeds were not enough for three replicates with at least 30 seeds in each germination test. SD, standard deviation.

**Table 3:** Maternal fertility of colchicine-treated ‘103-1’ (*Kalanchoe garambiensis* ‘Type 1’ × *K. nyikae*) could be restored based on observation of seed production and germination after open pollination.

Clone	Colchicine Concentration(mg∙L^−1^)	Mean (Open-Pollinated Seeds/Capsule, SD)	Seedling Number ^1^	Seed Germination(%) ^2^
‘103-1’	0	0 (0/28, 0.0) ^d^	- ^3^	-
‘103-1’-1	10,000	2.3 (25/11, 0.4) ^bc^	14	56 ^‡^
‘103-1’-2	10,000	0.5 (6/11, 0.5) ^cd^	4	66.7 ^‡^
‘103-1’-3	10,000	12.7 (38/3, 2.1) ^a^	36	94.7 ^‡^
‘103-1’-4	10,000	11.5 (69/6, 4.3) ^a^	56	81.2 ^‡^
‘103-1’-5	15,000	0 (0/3, 0.0) ^d^	-	-
‘103-1’-6	15,000	3 (18/6, 1.0) ^b^	9	50 ^‡^
‘103-1’-7	15,000	0 (0/12, 0.0) ^d^	-	-
‘103-1’-10	20,000	0 (0/13, 0.0) ^d^	-	-
‘103-1’-11	20,000	0 (0/12, 0.0) ^d^	-	-
‘103-1’-12	20,000	0 (0/7, 0.0) ^d^	-	-

^1^ Seedling number: total seedlings obtained from all collected seeds. ^2^ Germination percentage: at least 30 seeds and 3 repetitions were carried, but if seeds are not enough for repetitions, all collected seeds were germinated. ^3^ -, indicates not applicable. Different letters indicate significant differences between each other at *p* < 0.05 using one-way ANOVA with Tukey’s HSD test for seed number/capsule. ^‡^ total seed number <90, not applicable for statistical analysis because seeds were not enough for three replicates with at least 30 seeds in each germination test. SD, standard deviation.

**Table 4:** The fertility restored ‘103-1’ (*Kalanchoe garambiensis* ‘Type 1’ × *K. nyikae*) clones are able to produce seedling by crossing with *K. blossfeldiana* cultivar or backcrossing with *K. nyikae*.

Maternal Donor	Pollen Donor	Fruit Set (%)	Seed Number /Capsules	Seedling Number	Germination Percentage (%)
*K. nyikae*	‘103-1’-1	100 (2/2)	2 (4/2) ^†^	4	100 ^‡^
*K. blossfeldiana* ‘Ida’	‘103-1’-3	100 (4/4)	2.3 (9/4) ^†^	8	88.9 ^‡^

^†^, indicates not applicable for statistical analysis (capsule number <3 or no enough groups to compare). ^‡^ total seed number <90, not applicable for statistical analysis because seeds were not enough for three replicates with at least 30 seeds in each germination test.

**Table 5:** Chromosome number and ploidy level of studied *Kalanchoe* species.

Species	Chromosome Number and Ploidy Level	Basic Chromosome Number	References
*K. garambiensis* Kudo	2n = 68	x = 17	[33]
*K. blossfeldiana* von Poelln.	2n = 34 (origins) 2n = 68 (cultivars)	x = 17	[8,17]
*K. nyikae* Engler	2n = 68	x = 17	[17]
*K. lobata* R. Fern.	n.a. ^1^	n.a.	n.a.
*K. velutina* Welw.	2n = 34	x = 17	[34,35]
*K. sexangularis* N. E. Brown	2n = 34	x = 17	[34]
*K. longiflora* Schltr.	2n = 34	x = 17	[34,35]

^1^ n.a. indicates not available.

## Data Availability

The data presented in this study are available on request from the corresponding author.
